# HeRA: an atlas of enhancer RNAs across human tissues

**DOI:** 10.1093/nar/gkaa940

**Published:** 2020-10-29

**Authors:** Zhao Zhang, Wei Hong, Hang Ruan, Ying Jing, Shengli Li, Yaoming Liu, Jun Wang, Wenbo Li, Lixia Diao, Leng Han

**Affiliations:** Department of Biochemistry and Molecular Biology, McGovern Medical School at The University of Texas Health Science Center at Houston, Houston, TX 77030, USA; Department of Biochemistry and Molecular Biology, McGovern Medical School at The University of Texas Health Science Center at Houston, Houston, TX 77030, USA; Department of Biochemistry and Molecular Biology, McGovern Medical School at The University of Texas Health Science Center at Houston, Houston, TX 77030, USA; Department of Biochemistry and Molecular Biology, McGovern Medical School at The University of Texas Health Science Center at Houston, Houston, TX 77030, USA; Department of Biochemistry and Molecular Biology, McGovern Medical School at The University of Texas Health Science Center at Houston, Houston, TX 77030, USA; Department of Biochemistry and Molecular Biology, McGovern Medical School at The University of Texas Health Science Center at Houston, Houston, TX 77030, USA; Department of Pediatrics, McGovern Medical School at The University of Texas Health Science Center at Houston, Houston, TX 77030, USA; Department of Biochemistry and Molecular Biology, McGovern Medical School at The University of Texas Health Science Center at Houston, Houston, TX 77030, USA; Department of Bioinformatics and Computational Biology, The University of Texas MD Anderson Cancer Center, Houston, TX 77030, USA; Department of Biochemistry and Molecular Biology, McGovern Medical School at The University of Texas Health Science Center at Houston, Houston, TX 77030, USA; Center for Precision Health, The University of Texas Health Science Center at Houston, Houston, TX 77030, USA

## Abstract

Enhancer RNA (eRNA) is a type of long non-coding RNA transcribed from DNA enhancer regions. Despite critical roles of eRNA in gene regulation, the expression landscape of eRNAs in normal human tissue remains unexplored. Using numerous samples from the Genotype-Tissue Expression project, we characterized 45 411 detectable eRNAs and identified tens of thousands of associations between eRNAs and traits, including gender, race, and age. We constructed a co-expression network to identify millions of putative eRNA regulators and target genes across different tissues. We further constructed a user-friendly data portal, Human enhancer RNA Atlas (HeRA, https://hanlab.uth.edu/HeRA/). In HeRA, users can search, browse, and download the eRNA expression profile, trait-related eRNAs, and eRNA co-expression network by searching the eRNA ID, gene symbol, and genomic region in one or multiple tissues. HeRA is the first data portal to characterize eRNAs from 9577 samples across 54 human tissues and facilitates functional and mechanistic investigations of eRNAs.

## INTRODUCTION

An enhancer is a type of distal DNA regulatory element that couples with a promoter to organize an enhancer–promoter loop that initiates gene expression (1). As recent studies demonstrated that an enhancer can transcribe non-coding RNA, this element has been defined as enhancer RNA (eRNA) (2). Thousands of eRNAs have been reported across different human tissues (3), and eRNA has been shown to act as a marker for activated enhancers (4,5) and play critical roles in gene regulation (6). For example, eRNA can act as a scaffold to maintain the stability of the transcription complex (7,8). Growing evidence has suggested that eRNA expression is associated with multiple traits, characteristics, and diseases. For example, expression of the eRNA *OLMALINC* is associated with body weight by regulating the gene stearoyl-coenzyme A desaturase, which is related to serum triglyceride metabolism (9); and the expression of an eRNA is associated with autism spectrum disorders in the human brain by affecting the target gene expression (10). Biogenesis of eRNA is regulated by transcription factors (TFs), which are recruited to the DNA enhancer region to modulate chromatin accessibility and initiate eRNA transcription (6). For example, myogenic differentiation 1 (*MYOD1*) induces more than 16,000 eRNAs during myogenic differentiation (11), while estrogen receptor 1 (*ESR1*) induces thousands of eRNAs to maintain transcriptional circuitry in breast cancer (12). Furthermore, eRNA expression is critical in mediating the expression of target genes. For example, *NET1e* regulates the expression of oncogene neuroepithelial cell transforming 1 (*NET1*) in breast cancer to promote tumorigenesis (3), while *HPSEe* regulates the expression of heparanase (*HPSE*) to promote cancer invasion and metastasis (13). From these various associations with eRNAs, we sought to comprehensively investigate the expression landscape and co-expression network of eRNAs to facilitate our understanding of the mechanism of gene expression regulation and human phenotypes.

The Genotype-Tissue Expression (GTEx) project provides large numbers of RNA-seq samples and multiple traits across 54 human tissues (14). The Encyclopedia of DNA Elements (ENCODE) Project (15,16), Functional Annotation of the Mammalian Genome (FANTOM) Project (17), and Roadmap Epigenomics Project (18) provide comprehensive annotations of enhancers. By integrating these datasets, we characterized the expression landscape and regulatory network of eRNAs and their associations with different traits across human tissues. We further developed a comprehensive data portal, the Human enhancer RNA Atlas (HeRA), to benefit the research community.

## DATA COLLECTION AND PROCESSING

### eRNA annotation and quantification

**Figure 1.**
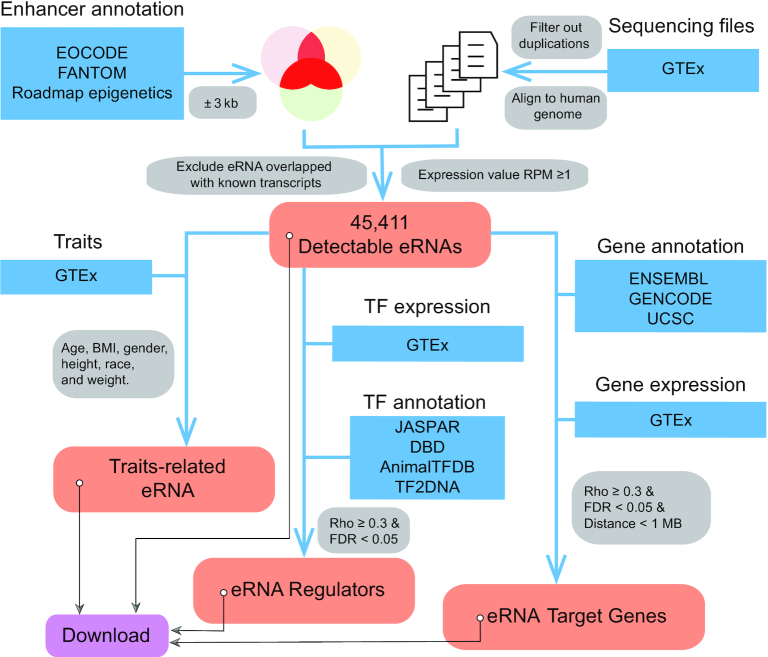
Computational framework for HeRA database. Blue blocks denote datasets used in this study. RNA-seq, traits and gene expression matrix were collected from GTEx. Enhancer annotation was collected from ENCODE, FANTOM and Roadmap Epigenetics Project. Transcription factors (TFs) were collected from AnimialTFDB, DBD, JASPAR and TF2DNA. Gene annotations were collected from ENSEMBL, GENCODE and UCSC. Red blocks denote processed data, including eRNA expression, traits, regulators and target genes. All processed data are available for downloading.

We collected the annotation of enhancers from ENCODE (Ensembl 87, http://dec2016.archive.ensembl.org/index.html) (15,16), FANTOM (https://fantom.gsc.riken.jp/5) (17) and the Roadmap Epigenomics Project (http://www.roadmapepigenomics.org) (18) (Supplementary Figure S1A). We transformed all annotations to hg19 version using Liftover (https://genome.ucsc.edu/cgi-bin/hgLiftOver) (19). We then integrated the eRNA annotations following the methods reported in our previous study (3). In brief, extending ±3kb around the middle enhancer loci, we screened enhancers that were annotated in at least two of these databases as a potential eRNA region. We excluded eRNA regions that overlapped with known transcripts (1 kb extension from both transcription start site and transcription end site), including coding genes and non-coding genes (e.g. tRNA, snoRNA and miRNAs) annotated in at least one of the following databases: Ensembl (http://dec2016.archive.ensembl.org/index.html) (15), UCSC (https://genome.ucsc.edu/index.html) (20) and GENCODE (https://www.gencodegenes.org/human/release_19.html) (21) (Figure 1). We collected RNA-seq files from GTEx (phs000424.v7.p2 on 26 July 2018, Supplementary Table S1) (14). We filtered out duplicate files by retaining the one with the largest number of reads. We then followed the methods described in previous GTEx publications (22–24), and mapped these reads to the human genome (hg19) using HISAT2 (http://daehwankimlab.github.io/hisat2/) (25), a SNP intolerant alignment approach. We obtained 9,577 bam files across 54 human tissues from 548 donors. We then characterized eRNA expression to calculate the number of reads for eRNA using SAMtools (26) and normalized the expression value using the reads per million (RPM) method (27). We considered only eRNAs with relatively high expression levels (RPM ≥ 1) as detectable eRNAs (Figure 1). We also used quantile normalization for all these eRNAs by R package preprocessCore (https://github.com/bmbolstad/preprocessCore). We further showed that co-expression analysis for trait-related eRNAs, eRNA-TF pairs and putative eRNA target genes, is highly consistent between RPM and quantile normalization in three tissues, including lung, liver, and brain cerebellum (Supplementary Figure S1B).

### Trait-related eRNAs

From the GTEx portal (https://www.gtexportal.org/home), we collected six traits: gender, race, age, height, weight, and body mass index (BMI) (14). We calculated the association between individual eRNA expression and each trait across tissues (28). We used the Student's *t* test to assess the statistical difference between eRNAs from male and female tissue donors and defined |fold change| > 1.5 and false discovery rate (FDR) < 0.05 as statistically significant. We used the analysis of variance (ANOVA) test to assess the statistical difference in eRNAs based on race and defined FDR <0.05 as significant. Only groups with ≥5 samples were included in the analyses by gender and race. We used Spearman's correlation to assess the statistical difference for the continuous traits of age, weight, height, and BMI, and defined |Rho| ≥ 0.3 and FDR < 0.05 as significant (Figure 1). All statistical analyses were analyzed by R, version 3.5.

### Putative regulators of eRNAs

We collected TFs from four TF data portals, AnimalTFDB (http://bioinfo.life.hust.edu.cn/AnimalTFDB/) (29), DBD (http://www.transcriptionfactor.org/) (30), JASPAR (http://jaspar.genereg.net/) (31) and TF2DNA (http://www.fiserlab.org/tf2dna_db/) (32), and retained TFs that were annotated in at least one of these databases (Supplementary Figure S2A). The expression matrix of TFs in human tissues was obtained from the GTEx portal. We then identified putative regulators of eRNAs based on the co-expression between eRNA and TF across tissues. Co-expression showing Spearman's correlation Rho ≥ 0.3 and FDR < 0.05 was considered to be significant. Furthermore, we screened potential TF binding sites (TFBS) to validate these eRNA–TF pairs. We collected TFBS based on ChIP-seq datasets from ENCODE project (https://www.encodeproject.org/) (33), and mapped them to those eRNAs co-expressed with TFs accordingly. Several TFs (e.g., CTCF, EP300, and RUNX3) have relatively high TFBS evidences for eRNA–TF pairs (>90%, Supplementary Figure S2B and Table S2). For those TFs with low percentage evidences, we speculated that this is due to the limited number of ChIP-seq experiments in ENCODE, that the majority of them are examined in ≤5 tissues/cell lines (34). We also performed GO enrichment by DAVID online tool (https://david.ncifcrf.gov/) (35) and observed that top 30% TFs with higher/lower TFBS discovery rates were both enriched in transcription related modules (Supplementary Figure S2C). We will update our data portal when new ChIP-seq data released by ENCODE or other consortiums.

### Putative eRNA target genes

We collected gene annotations from ENSEMBL (http://dec2016.archive.ensembl.org/index.html) (15), GENOCODE (https://www.gencodegenes.org/human/release_19.html) (21) and UCSC (https://genome.ucsc.edu/index.html) (20) and merged them. We collected the expression matrix of these genes across human tissues from the GTEx portal. We identified putative eRNA target genes based on relatively close distance (≤1MB) and significant co-expression (Spearman's correlation Rho ≥ 0.3 and FDR < 0.05) in each tissue. We also performed random sampling (10 000 pairs) of interchromosomal pairs, and observed that the Rho is around 0.02 and FDR is around 0.3 (Supplementary Figure S3A), which is much lower than our cutoff (Rho > 0.3 and FDR < 0.05), suggesting that our cutoff is reliable. The strength of eRNA-target gene associations is inversely dependent on the distance (Supplementary Figure S3B), which is consistent with previous studies (e.g. FAMTOM5 and ENCODE) (36,37). In addition, non-strand specific RNA-seq collected in GTEx dataset is not appropriate to identify eRNA transcribed from antisense strand (https://www.gtexportal.org/home/documentationPage) (38). Therefore, we filtered out putative associations in which the eRNA was located in the intronic region of the target gene (3,39).

### Web design of HeRA

We developed the interface of HeRA using the Bootstrap 4 framework, which includes HTML, CSS and JavaScript code (http://getbootstrap.com/). We designed the HeRA website using Python 2.7.2, with the Django web-framework. To perform data analyses and data plotting, we used R, version 3.5.3. We provide all the data, including eRNA expression, trait-related eRNAs, eRNA regulators, and eRNA target genes, for browsing and querying in each module of HeRA.

## DATABASE CONTENT AND USAGE

### Sample summary and expression landscape of human eRNAs

We collected 9577 samples across 54 normal human tissues, ranging from 5 in cervix - endocervix to 477 in muscle - skeletal, with median of 141 in liver and artery—coronary (Supplementary Table S1). In these tissues, we identified 45 411 detectable eRNAs in total. The number of detectable eRNAs in different tissues ranged from 2069 in heart—left ventricle to 14 232 in testis, with median of 4629 in esophagus—mucosa (Supplementary Table S1).

### Data searching and browsing for four modules

**Figure 2.**
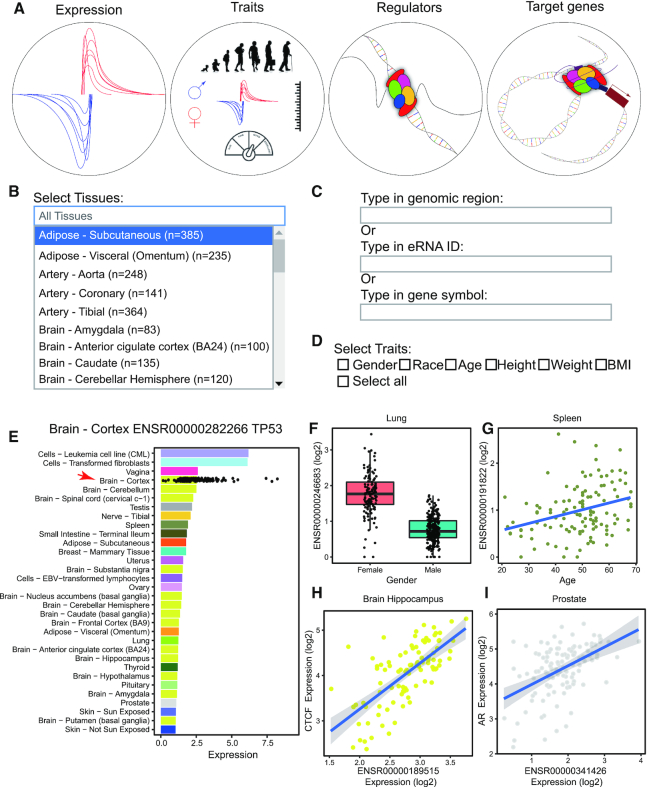
Overview of HeRA database. (**A**) Four modules of HeRA: expression, traits, regulators, and targets. (**B**) Interface of search boxes for tissues in each module. Users can select one or more tissues for querying. Default is all tissues. (**C**) Interface of search boxes for eRNA in each module. Users may query through the genomic region, eRNA ID, or gene symbol. (**D**) Trait selection function in the traits module. Users can select one or more traits for querying. (**E**) Mean expression of ENSR00000282266 across tissues (bar) and in each individual sample of brain—cortex (dot). (**F**) Differential expression of ENSR00000246683 in lung tissue between females and males. (**G**) Significant correlation of ENSR00000191822 with age in splenic tissue. (**H**) Co-expression of ENSR00000189515 and putative regulator *CTCF* in brain—hippocampus. (**I**) Co-expression of ENSR00000341426 and putative target gene *AR* in prostate tissue.

We developed HeRA for browsing, searching, and downloading eRNA expression and trait-related eRNAs and the co-expression network. HeRA consists of four modules: ***expression***, ***traits***, ***regulators*** and ***target genes*** (Figure 2A). In each module, we designed two boxes: a tissue selection box, in which users can select one or more tissues for querying (Figure 2B); and an eRNA search box, in which users can search eRNAs through the genomic region, eRNA ID or gene symbol for querying (Figure 2C). For the eRNA search box, typing in a genomic region eRNA will query all eRNAs that overlap with that genomic region; typing in an eRNA ID will query the unique eRNA with this ID; and typing in a gene symbol will query all eRNAs located ±1 MB around the transcription start site of the selected gene. In the module ***traits***, we supply an additional box for trait selection, in which users can select one or more traits for querying (Figure 2D).

In the ***expression*** module, users can search the eRNA expression landscape across tissues. For example, ENSR00000282266, the eRNA located on chr17:6785018–6791019 within 1 MB of tumor protein P53 (*TP53*), is detectable in 32 tissues, and the mean expression value in the brain - cortex is 2.55 (Figure 2E), suggesting this might be a novel eRNA for P53. We also provided the ‘Download’ button to allow users to download sequence in FASTA format for the queried eRNAs. In the ***traits*** module, users can search trait-related eRNAs across tissues. For example, ENSR00000246683, the eRNA located on chrX:53211191–53217192 and within 1 MB of lysine demethylase 5C (*KDM5C*), and sourced from lung tissue, showed differential expression between male and female tissue donors (Student's *t* test, fold-change = —1.99 and FDR < 2.2 × 10^−16^, Figure 2F). This is consistent with expression alternation of *KDM5C* between male and female in lung tissue (Supplementary Figure S4A). ENSR00000191822, the eRNA located on chr6:384543–390544 within 1 MB of interferon regulatory factor 4 (*IRF4*), and sourced from splenic tissue, showed significant association with the age of the tissue donor (Spearman correlation Rho = 0.32, FDR = 0.028, Figure 2G). This is consistent with expression correlation of *IRF4* and age in spleen tissue (Supplementary Figure S4B). In the ***regulators*** module, users can search putative regulators (e.g. TFs) of each eRNA across tissues. We used significant co-expression (Rho ≥ 0.3 and FDR < 0.05) between eRNA and TFs to identify the putative regulatory relationship. For example, the expression of ENSR00000189515 is significantly correlated with CCCTC-binding factor (*CTCF*), a TF, in the brain – hippocampus (Rho = 0.69, FDR = 1.90 × 10^−12^), which suggests that *CTCF* is a potential regulator of ENSR00000189515 (Figure 2H). In the ***target genes*** module, users can search putative targets of eRNAs across tissues. We used close distance (within 1 MB between eRNA and gene) and significant co-expression (Rho ≥ 0.3 and FDR < 0.05) to identify putative target genes. For example, ENSR00000341426 is within 1 MB of the androgen receptor (*AR*), and significantly correlates with *AR* in prostate tissue (distance = 474 778 bp, Rho = 0.46, FDR = 1.39 × 10^−5^), which suggests that ENSR00000341426 may regulate the expression of *AR* in the prostate (Figure 2I).

### Data download and maintenance

We provide download functions for all four modules of HeRA. In the ***expression*** module, users can download the image file as a PDF and table file in csv format and sequence file in FASTA format for a queried eRNA in each tissue. The PDF file is consistent with the displayed image in the ***expression*** module (e.g. Figure 2E) and the table file includes the eRNA expression in each sample. In the ***traits*** module, we provide a table file for all queried results and a PDF for each queried trait (e.g. Figure 2F, G). For the ***regulator*** module and ***target genes***module, users can download an image for each eRNA-gene pair as a PDF (e.g. Figure 2H, I). In addition, we provide a download page (https://hanlab.uth.edu/HeRA/download) to allow users to download the whole dataset, including expression, co-expression, and sequence for customized analysis.

## SUMMARY AND FUTURE DIRECTIONS

By integrating multiple datasets, including ENCODE, FANTOM, and GTEx, we systematically quantified the expression of eRNA, trait-related eRNAs, putative eRNA regulators, and putative eRNA target genes across 54 normal human tissues. We developed a user-friendly data portal, HeRA, through which users can query, browse and download eRNA and eRNA-related events across tissues. HeRA can serve as a valuable resource for understanding the expression, association with traits, biogenesis and targets of eRNAs in human tissues. In this data resource, we followed the computational pipeline introduced by GTEx, that we used the SNP intolerant alignment. It is possible that reference mapping bias can lead to false positive co-expression patterns. Further studies are necessary to assess the impact of reference mapping bias on the co-expression studies, and whether more computationally expensive allele specific RNA-seq alignment is necessary or not. Furthermore, it is a long-known widespread consensus in the human genetic and systems biology field that for larger scale association studies, such as GWAS, eQTL and co-expression network analysis (3,40–43), RPM/RPKM/FPKM normalizations can be vulnerable to confounding artifacts. There have been a number of proposed solutions, including principal component normalization (44), that can be used to reveal sources of confounders and build co-expression network. Furthermore, it might be interesting to investigate the cross-species conservation, despite the fact that it is still challenging to characterize the cross-species conservation of ncRNAs (45), especially for the newly emerging eRNAs. We will conduct above analyses to further refine HeRA data portal in the future. In summary, we comprehensively characterized the eRNA expression landscape across human tissues and provide a useful data portal for investigating the function and underlying mechanism of eRNAs. We will continue to update this useful resource along with the booming number of related samples to benefit the research community.

## Supplementary Material

gkaa940_Supplemental_File

## References

[1] Khoury G., Gruss P. Enhancer elements. Cell. 1983; 33:313–314. 6305503 10.1016/0092-8674(83)90410-5

[2] de Santa F., Barozzi I., Mietton F., Ghisletti S., Polletti S., Tusi B.K., Muller H., Ragoussis J., Wei C.L., Natoli G. A large fraction of extragenic RNA Pol II transcription sites overlap enhancers. PLoS Biol. 2010; 8:e1000384. 20485488 10.1371/journal.pbio.1000384 PMC2867938

[3] Zhang Z., Lee J.H., Ruan H., Ye Y., Krakowiak J., Hu Q., Xiang Y., Gong J., Zhou B., Wang L. et al. Transcriptional landscape and clinical utility of enhancer RNAs for eRNA-targeted therapy in cancer. Nat. Commun. 2019; 10:4562. 31594934 10.1038/s41467-019-12543-5 PMC6783481

[4] Hah N., Murakami S., Nagari A., Danko C.G., Lee Kraus W. Enhancer transcripts mark active estrogen receptor binding sites. Genome Res. 2013; 23:1210–1223. 23636943 10.1101/gr.152306.112 PMC3730096

[5] Mikhaylichenko O., Bondarenko V., Harnett D., Schor I.E., Males M., Viales R.R., Furlong E.E.M. The degree of enhancer or promoter activity is reflected by the levels and directionality of eRNA transcription. Genes Dev. 2018; 32:42–57. 29378788 10.1101/gad.308619.117 PMC5828394

[6] Li W., Notani D., Rosenfeld M.G. Enhancers as non-coding RNA transcription units: Recent insights and future perspectives. Nat. Rev. Genet. 2016; 17:207–223. 26948815 10.1038/nrg.2016.4

[7] Hsieh C.L., Fei T., Chen Y., Li T., Gao Y., Wang X., Sun T., Sweeney C.J., Lee G.S.M., Chen S. et al. Enhancer RNAs participate in androgen receptor-driven looping that selectively enhances gene activation. Proc. Natl. Acad. Sci. U.S.A. 2014; 111:7319–7324. 24778216 10.1073/pnas.1324151111 PMC4034202

[8] Tsai P.F., Dell’Orso S., Rodriguez J., Vivanco K.O., Ko K.D., Jiang K., Juan A.H., Sarshad A.A., Vian L., Tran M. et al. A muscle-specific enhancer RNA mediates cohesin recruitment and regulates transcription in trans. Mol. Cell. 2018; 71:129–141. 29979962 10.1016/j.molcel.2018.06.008 PMC6082425

[9] Benhammou J.N., Ko A., Alvarez M., Kaikkonen M.U., Rankin C., Garske K.M., Padua D., Bhagat Y., Kaminska D., Kärjä V. et al. Novel lipid long intervening noncoding RNA, oligodendrocyte maturation‐associated long intergenic noncoding RNA, regulates the liver steatosis gene stearoyl‐coenzyme A desaturase as an enhancer RNA. Hepatol. Commun. 2019; 3:1356–1372. 31592021 10.1002/hep4.1413 PMC6771395

[10] Yao P., Lin P., Gokoolparsadh A., Assareh A., Thang M.W.C., Voineagu I. Coexpression networks identify brain region-specific enhancer RNAs in the human brain. Nat. Neurosci. 2015; 18:1168–1174. 26167905 10.1038/nn.4063

[11] Zhao Y., Zhou J., He L., Li Y., Yuan J., Sun K., Chen X., Bao X., Esteban M.A., Sun H. et al. MyoD induced enhancer RNA interacts with hnRNPL to activate target gene transcription during myogenic differentiation. Nat. Commun. 2019; 10:5787. 31857580 10.1038/s41467-019-13598-0 PMC6923398

[12] Li W., Notani D., Ma Q., Tanasa B., Nunez E., Chen A.Y., Merkurjev D., Zhang J., Ohgi K., Song X. et al. Functional roles of enhancer RNAs for oestrogen-dependent transcriptional activation. Nature. 2013; 498:516–520. 23728302 10.1038/nature12210 PMC3718886

[13] Jiao W., Chen Y., Song H., Li D., Mei H., Yang F., Fang E., Wang X., Huang K., Zheng L. et al. HPSE enhancer RNA promotes cancer progression through driving chromatin looping and regulating hnRNPU/p300/EGR1/HPSE axis. Oncogene. 2018; 37:2728–2745. 29511351 10.1038/s41388-018-0128-0

[14] Lonsdale J., Thomas J., Salvatore M., Phillips R., Lo E., Shad S., Hasz R., Walters G., Garcia F., Young N. et al. The Genotype-Tissue Expression (GTEx) project. Nat. Genet. 2013; 45:580–585. 23715323 10.1038/ng.2653 PMC4010069

[15] Yates A.D., Achuthan P., Akanni W., Allen J., Allen J., Alvarez-Jarreta J., Amode M.R., Armean I.M., Azov A.G., Bennett R. et al. Ensembl 2020. Nucleic. Acids. Res. 2020; 48:D682–D688. 31691826 10.1093/nar/gkz966 PMC7145704

[16] Luo Y., Hitz B.C., Gabdank I., Hilton J.A., Kagda M.S., Lam B., Myers Z., Sud P., Jou J., Lin K. et al. New developments on the Encyclopedia of DNA Elements (ENCODE) data portal. Nucleic Acids Res. 2020; 48:D882–D889. 31713622 10.1093/nar/gkz1062 PMC7061942

[17] Lizio M., Abugessaisa I., Noguchi S., Kondo A., Hasegawa A., Hon C.C., De Hoon M., Severin J., Oki S., Hayashizaki Y. et al. Update of the FANTOM web resource: Expansion to provide additional transcriptome atlases. Nucleic Acids Res. 2019; 47:D752–D758. 30407557 10.1093/nar/gky1099 PMC6323950

[18] Chadwick L.H. The NIH roadmap epigenomics program data resource. Epigenomics. 2012; 4:317–324. 22690667 10.2217/epi.12.18 PMC3381455

[19] Haeussler D., Zweig A.S., Tyner C., Speir M.L., Rosenbloom K.R., Raney B.J., Lee C.M., Lee B.T., Hinrichs A.S., Gonzalez J.N. et al. The UCSC Genome Browser Daabase: 2019 update. Nucleic Acids Res. 2019; 47:D853–D858. 30407534 10.1093/nar/gky1095 PMC6323953

[20] Lee C.M., Barber G.P., Casper J., Clawson H., Diekhans M., Gonzalez J.N., Hinrichs A.S., Lee B.T., Nassar L.R., Powell C.C. et al. UCSC Genome Browser enters 20th year. Nucleic. Acids. Res. 2020; 48:D756–D761. 31691824 10.1093/nar/gkz1012 PMC7145642

[21] Frankish A., Diekhans M., Ferreira A.M., Johnson R., Jungreis I., Loveland J., Mudge J.M., Sisu C., Wright J., Armstrong J. et al. GENCODE reference annotation for the human and mouse genomes. Nucleic Acids Res. 2019; 47:D766–D773. 30357393 10.1093/nar/gky955 PMC6323946

[22] Saha A., Kim Y., Gewirtz A.D.H., Jo B., Gao C., McDowell I.C., Engelhardt B.E., Battle A. Co-expression networks reveal the tissue-specific regulation of transcription and splicing. Genome Res. 2017; 27:1843–1858. 29021288 10.1101/gr.216721.116 PMC5668942

[23] Pierson E., Koller D., Battle A., Mostafavi S. Sharing and specificity of co-expression networks across 35 human tissues. PLoS Comput. Biol. 2015; 11:e1004220. 25970446 10.1371/journal.pcbi.1004220 PMC4430528

[24] Yizhak K., Aguet F., Kim J., Hess J.M., Kübler K., Grimsby J., Frazer R., Zhang H., Haradhvala N.J., Rosebrock D. et al. RNA sequence analysis reveals macroscopic somatic clonal expansion across normal tissues. Science. 2019; 364:eaaw0726. 31171663 10.1126/science.aaw0726 PMC7350423

[25] Kim D., Langmead B., Salzberg S.L. HISAT: a fast spliced aligner with low memory requirements. Nat. Methods. 2015; 12:357–360. 25751142 10.1038/nmeth.3317 PMC4655817

[26] Li H., Handsaker B., Wysoker A., Fennell T., Ruan J., Homer N., Marth G., Abecasis G., Durbin R. The Sequence Alignment/Map format and SAMtools. Bioinformatics. 2009; 25:2078–2079. 19505943 10.1093/bioinformatics/btp352 PMC2723002

[27] Mortazavi A., Williams B.A., McCue K., Schaeffer L., Wold B. Mapping and quantifying mammalian transcriptomes by RNA-Seq. Nat. Methods. 2008; 5:621–628. 18516045 10.1038/nmeth.1226 PMC13303166

[28] Hong W., Ruan H., Zhang Z., Ye Y., Liu Y., Li S., Jing Y., Zhang H., Diao L., Liang H. et al. APAatlas: decoding alternative polyadenylation across human tissues. Nucleic Acids Res. 2020; 48:D34–D39. 31586392 10.1093/nar/gkz876 PMC6943053

[29] Hu H., Miao Y.R., Jia L.H., Yu Q.Y., Zhang Q., Guo A.Y. AnimalTFDB 3.0: a comprehensive resource for annotation and prediction of animal transcription factors. Nucleic. Acids. Res. 2019; 47:D33–D38. 30204897 10.1093/nar/gky822 PMC6323978

[30] Kummerfeld S.K. DBD: a transcription factor prediction database. Nucleic Acids Res. 2006; 34:D74–D81. 16381970 10.1093/nar/gkj131 PMC1347493

[31] Fornes O., Castro-Mondragon J.A., Khan A., Van Der Lee R., Zhang X., Richmond P.A., Modi B.P., Correard S., Gheorghe M., Baranašić D. et al. JASPAR 2020: update of the open-access database of transcription factor binding profiles. Nucleic Acids Res. 2020; 48:D87–D92. 31701148 10.1093/nar/gkz1001 PMC7145627

[32] Pujato M., Kieken F., Skiles A.A., Tapinos N., Fiser A. Prediction of DNA binding motifs from 3D models of transcription factors; identifying TLX3 regulated genes. Nucleic Acids Res. 2014; 42:13500–13512. 25428367 10.1093/nar/gku1228 PMC4267649

[33] Moore J.E., Purcaro M.J., Pratt H.E., Epstein C.B., Shoresh N., Adrian J., Kawli T., Davis C.A., Dobin A., Kaul R. et al. Expanded encyclopaedias of DNA elements in the human and mouse genomes. Nature. 2020; 583:699–710. 32728249 10.1038/s41586-020-2493-4 PMC7410828

[34] Vierstra J., Lazar J., Sandstrom R., Halow J., Lee K., Bates D., Diegel M., Dunn D., Neri F., Haugen E. et al. Global reference mapping of human transcription factor footprints. Nature. 2020; 583:729–736. 32728250 10.1038/s41586-020-2528-x PMC7410829

[35] Huang D.W., Sherman B.T., Lempicki R.A. Systematic and integrative analysis of large gene lists using DAVID bioinformatics resources. Nat. Protoc. 2009; 4:44–57. 19131956 10.1038/nprot.2008.211

[36] Andersson R., Gebhard C., Miguel-Escalada I., Hoof I., Bornholdt J., Boyd M., Chen Y., Zhao X., Schmidl C., Suzuki T. et al. An atlas of active enhancers across human cell types and tissues. Nature. 2014; 507:455–461. 24670763 10.1038/nature12787 PMC5215096

[37] Moore J.E., Purcaro M.J., Pratt H.E., Epstein C.B., Shoresh N., Adrian J., Kawli T., Davis C.A., Dobin A., Kaul R. et al. Expanded encyclopaedias of DNA elements in the human and mouse genomes. Nature. 2020; 583:699–710. 32728249 10.1038/s41586-020-2493-4 PMC7410828

[38] Aguet F., Brown A.A., Castel S.E., Davis J.R., He Y., Jo B., Mohammadi P., Park Y.S., Parsana P., Segrè A.V. et al. Genetic effects on gene expression across human tissues. Nature. 2017; 550:204–213. 29022597 10.1038/nature24277 PMC5776756

[39] Chen H., Li C., Peng X., Zhou Z., Weinstein J.N., Caesar-Johnson S.J., Demchok J.A., Felau I., Kasapi M., Ferguson M.L. et al. A pan-cancer analysis of enhancer expression in nearly 9000 patient samples. Cell. 2018; 173:386–399. 29625054 10.1016/j.cell.2018.03.027 PMC5890960

[40] Gong J., Mei S., Liu C., Xiang Y., Ye Y., Zhang Z., Feng J., Liu R., Diao L., Guo A.Y. et al. PancanQTL: systematic identification of cis -eQTLs and trans -eQTLs in 33 cancer types. Nucleic Acids Res. 2018; 46:D971–D976. 29036324 10.1093/nar/gkx861 PMC5753226

[41] Joung J., Engreitz J.M., Konermann S., Abudayyeh O.O., Verdine V.K., Aguet F., Gootenberg J.S., Sanjana N.E., Wright J.B., Fulco C.P. et al. Genome-scale activation screen identifies a lncRNA locus regulating a gene neighbourhood. Nature. 2017; 548:343–346. 28792927 10.1038/nature23451 PMC5706657

[42] Aguet F., Brown A.A., Castel S.E., Davis J.R., He Y., Jo B., Mohammadi P., Park Y.S., Parsana P., Segrè A.V. et al. Genetic effects on gene expression across human tissues. Nature. 2017; 550:204–213. 29022597 10.1038/nature24277 PMC5776756

[43] Tian D., Wang P., Tang B., Teng X., Li C., Liu X., Zou D., Song S., Zhang Z. GWAS Atlas: a curated resource of genome-wide variant-trait associations in plants and animals. Nucleic Acids Res. 2020; 48:D927–D932. 31566222 10.1093/nar/gkz828 PMC6943065

[44] Parsana P., Ruberman C., Jaffe A.E., Schatz M.C., Battle A., Leek J.T. Addressing confounding artifacts in reconstruction of gene co-expression networks. Genome Biol. 2019; 20:94. 31097038 10.1186/s13059-019-1700-9 PMC6521369

[45] Diederichs S. The four dimensions of noncoding RNA conservation. Trends Genet. 2014; 30:121–123. 24613441 10.1016/j.tig.2014.01.004

