# Supplementary material for: HeRA: an atlas of enhancer RNAs across human tissues
Source: Nucleic Acids Res. 2020 Oct 29;49(D1):D932–8. doi: 10.1093/nar/gkaa940 (PMC7779069; doi:10.1093/nar/gkaa940)
Supplement: gkaa940_Supplemental_File [file gkaa940_supplemental_file.pdf]

Figure S1

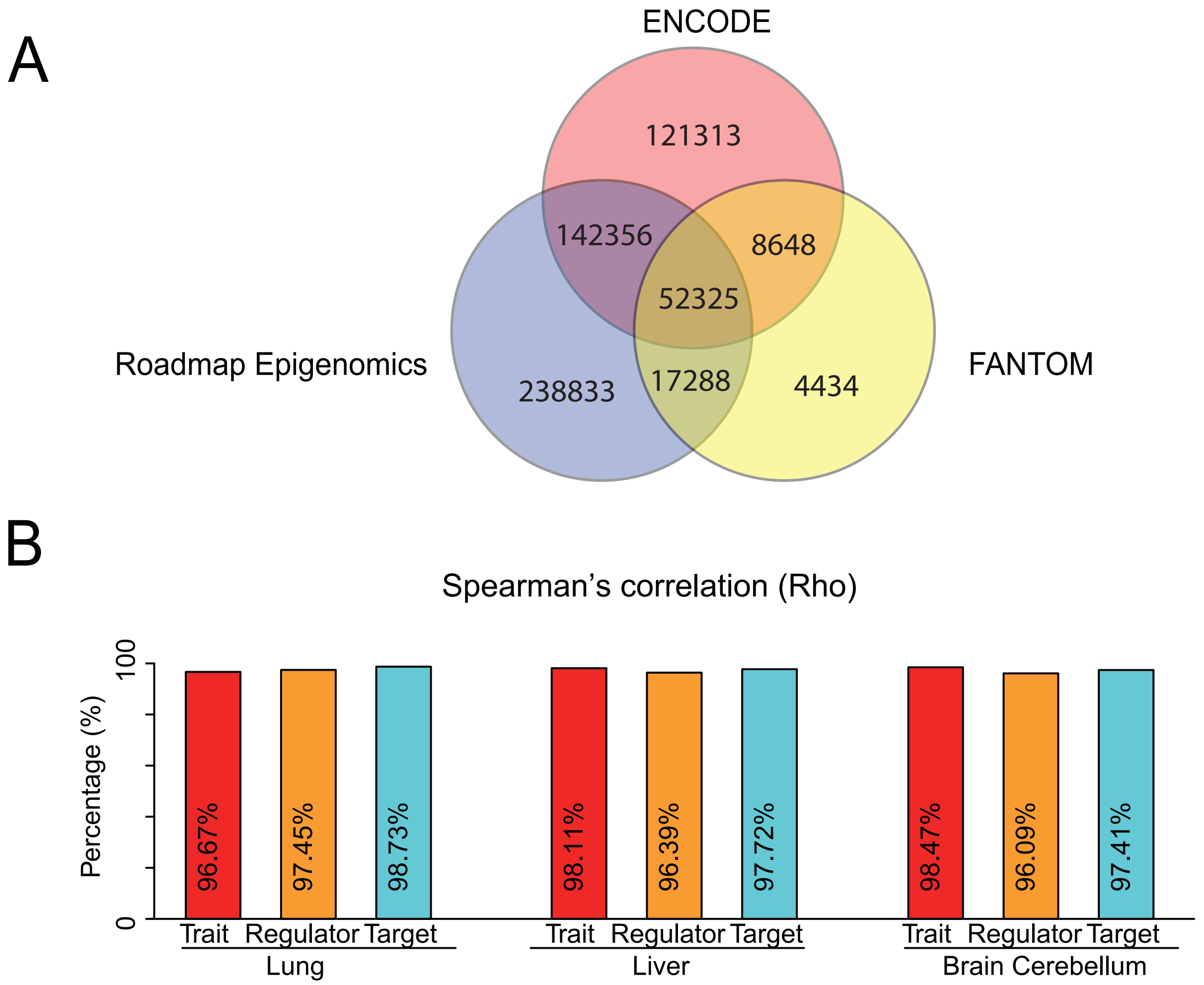

Figure S1. (A) Number of enhancer annotated in ENCODE, FANTOM and Roadmap Epigenomics. (B) Percentage of consistent instances between RPM and quantile normalized expression in three tissues.

Figure S2

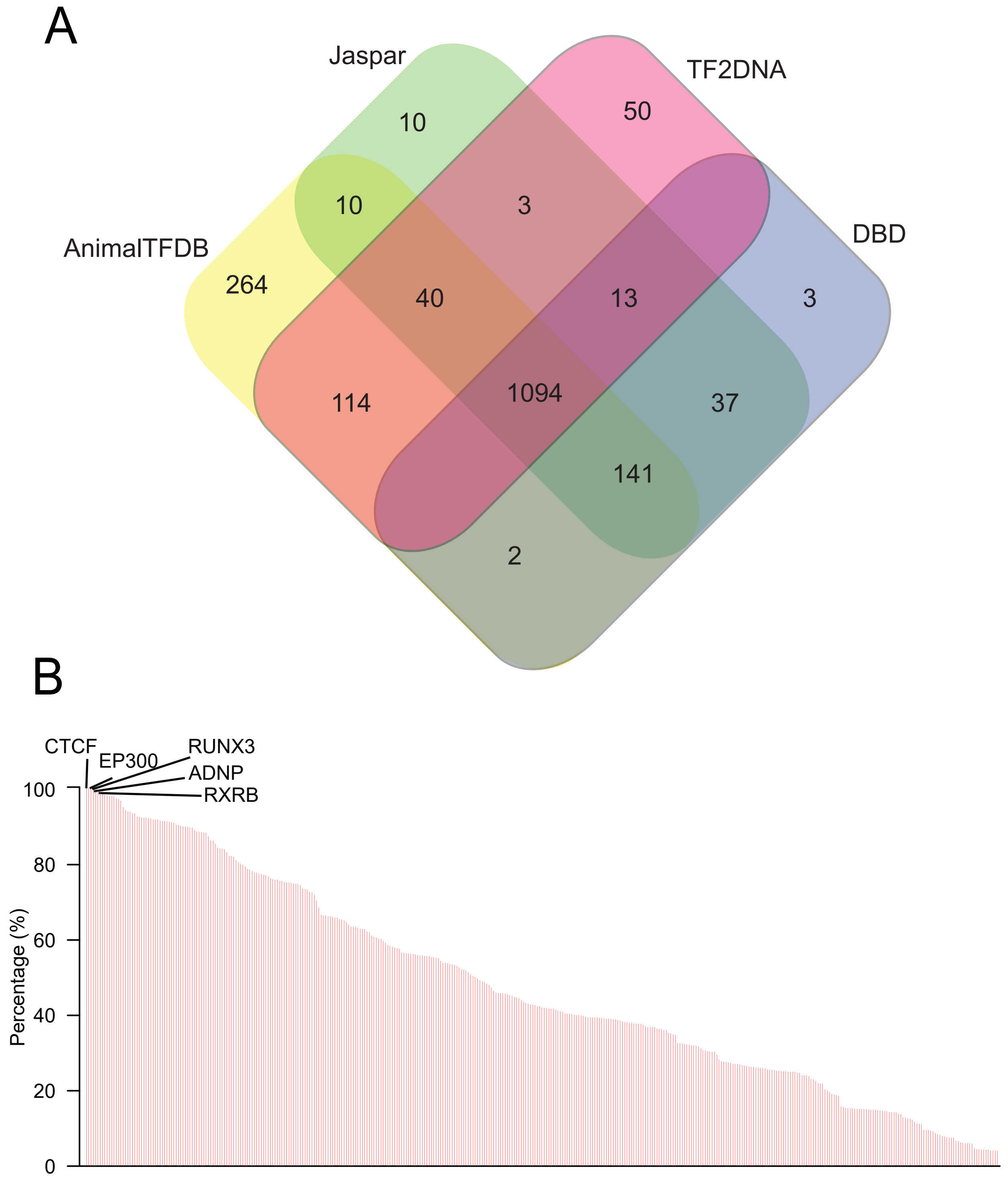
A
Jaspar
TF2DNA
AnimalTFDB
DBD
10
50
10
3
264
40
13
3
114
1094
37
141
2
B
CTCF
EP300
RUNX3
ADNP
RXRB
Percentage (%)
100
80
60
40
20
0

C

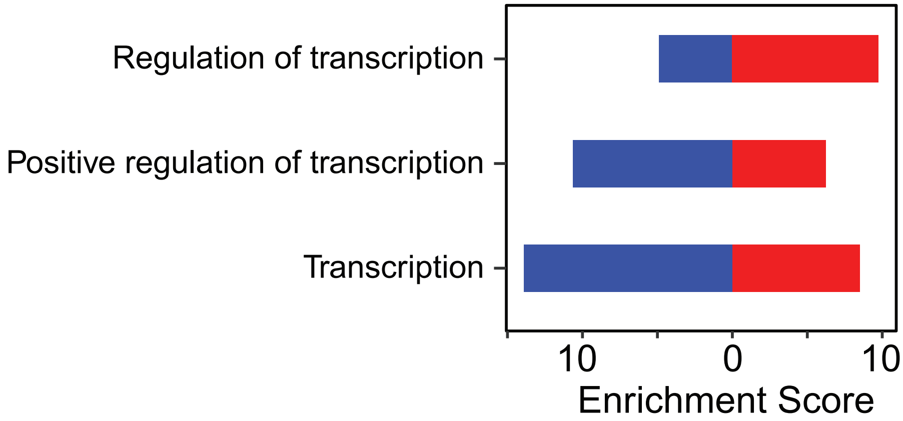

Figure S2. (A) Number of TF annotated in Jaspar, AnimalTFDB, TF2DNA and DBD. (B) Percentage of eRNA-TF pairs with TFBS evidences. X-axis denotes TFs. Y-axis denotes to the percentage of co-expressed eRNA-TF pairs for each TF. (C) Top 3 enrichment GO items of top 30% TFs with higher (red)/lower (blue) TFBS discovery rates.

Figure S3

A

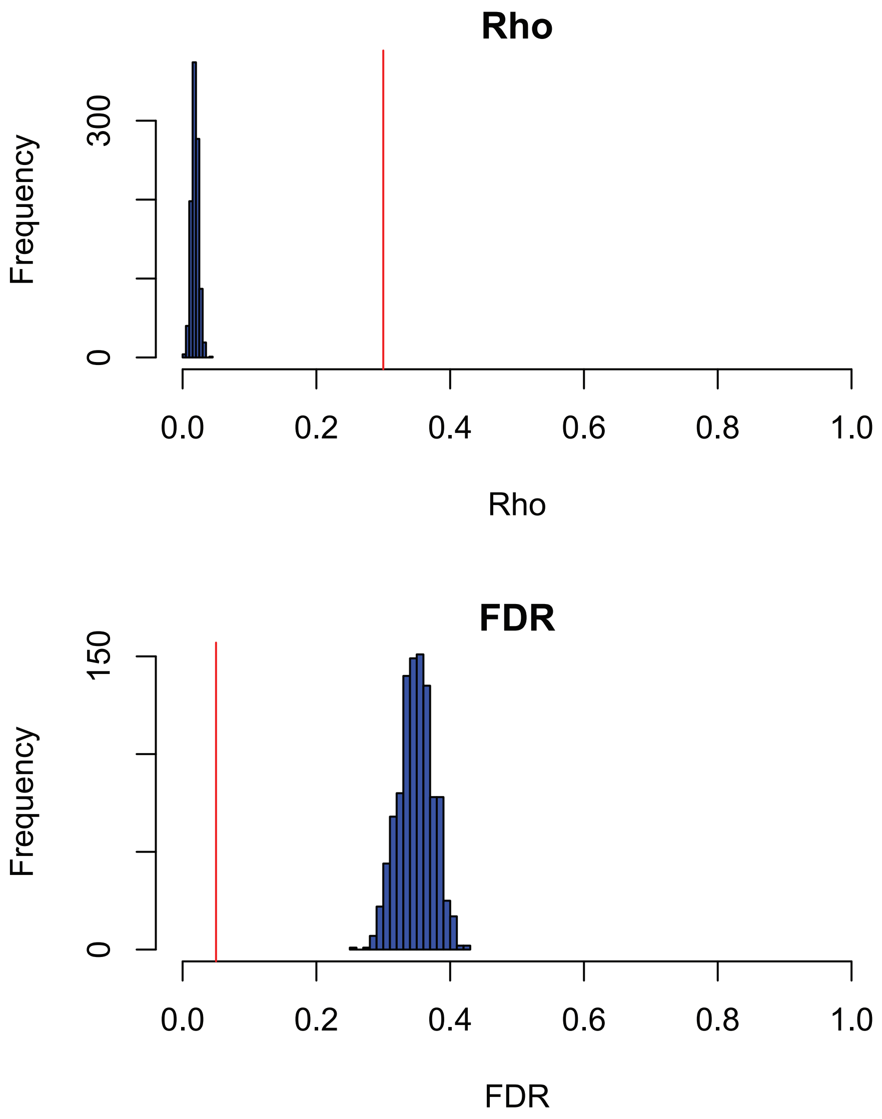
Rho
Frequency
0
300
0.0
0.2
0.4
0.6
0.8
1.0
Rho
FDR
Frequency
0
150
0.0
0.2
0.4
0.6
0.8
1.0
FDR

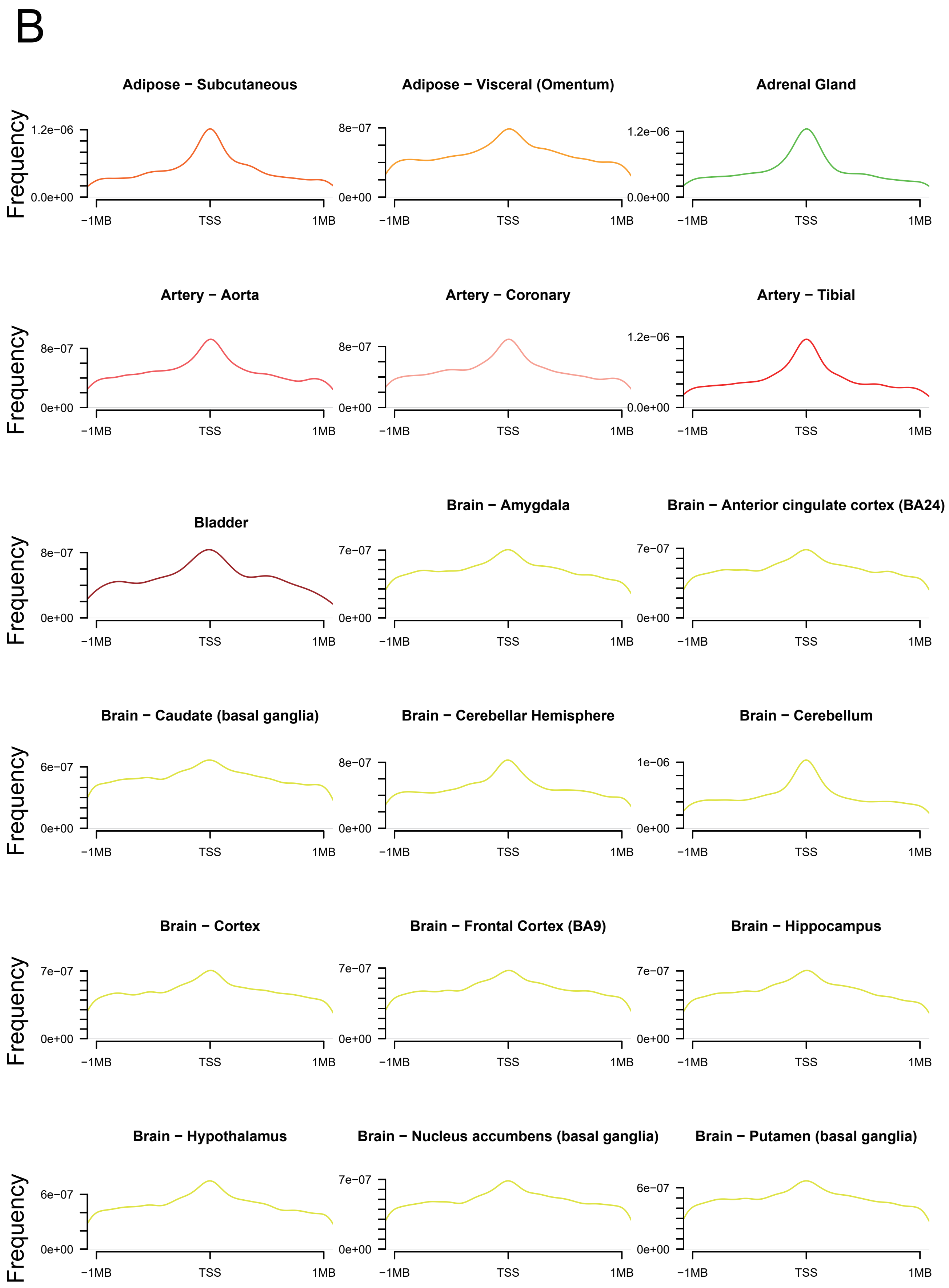
B
Adipose − Subcutaneous
Adipose − Visceral (Omentum)
Adrenal Gland
Artery − Aorta
Artery − Coronary
Artery − Tibial
Bladder
Brain − Amygdala
Brain − Anterior cingulate cortex (BA24)
Brain − Caudate (basal ganglia)
Brain − Cerebellar Hemisphere
Brain − Cerebellum
Brain − Cortex
Brain − Frontal Cortex (BA9)
Brain − Hippocampus
Brain − Hypothalamus
Brain − Nucleus accumbens (basal ganglia)
Brain − Putamen (basal ganglia)
Frequency
−1MB
TSS
1MB

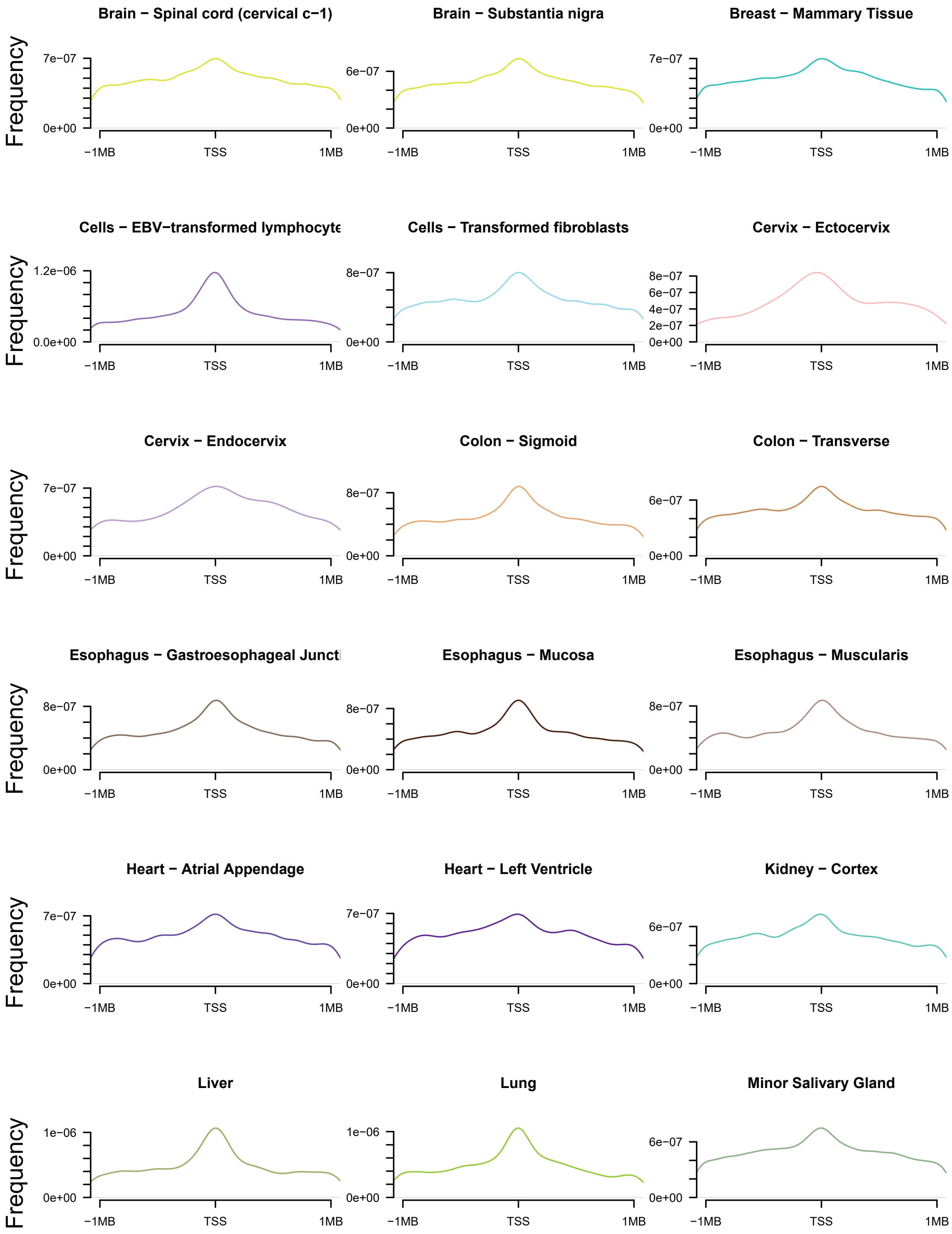
Brain − Spinal cord (cervical c−1)
Brain − Substantia nigra
Breast − Mammary Tissue
Cells − EBV−transformed lymphocyte
Cells − Transformed fibroblasts
Cervix − Ectocervix
Cervix − Endocervix
Colon − Sigmoid
Colon − Transverse
Esophagus − Gastroesophageal Juncti
Esophagus − Mucosa
Esophagus − Muscularis
Heart − Atrial Appendage
Heart − Left Ventricle
Kidney − Cortex
Liver
Lung
Minor Salivary Gland
Frequency
−1MB
TSS
1MB
7e−07
6e−07
8e−07
1.2e−06
0.0e+00
0e+00
8e−07
6e−07
4e−07
2e−07
1e−06

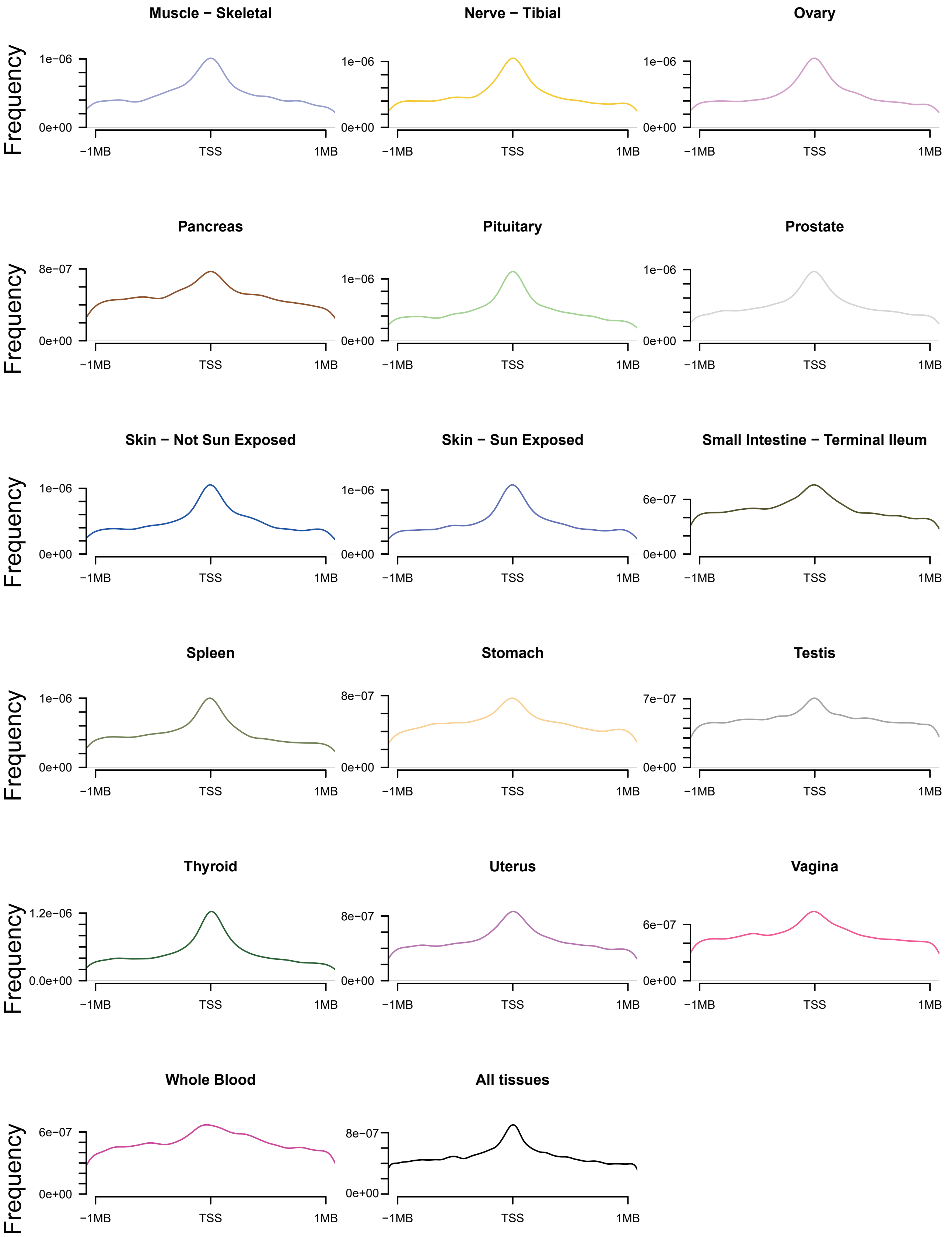

Figure S3. (A) Distribution of correlation (blue histogram) for interchromosomal eRNA-target gene pair of Rho (upper panel) and FDR (bottom panel) pair. Red line indicates our cutoff for eRNA-target gene pair. (B) Distribution between eRNA-gene association and distances across tissues. Y-axis denotes frequency.

Figure S4

A

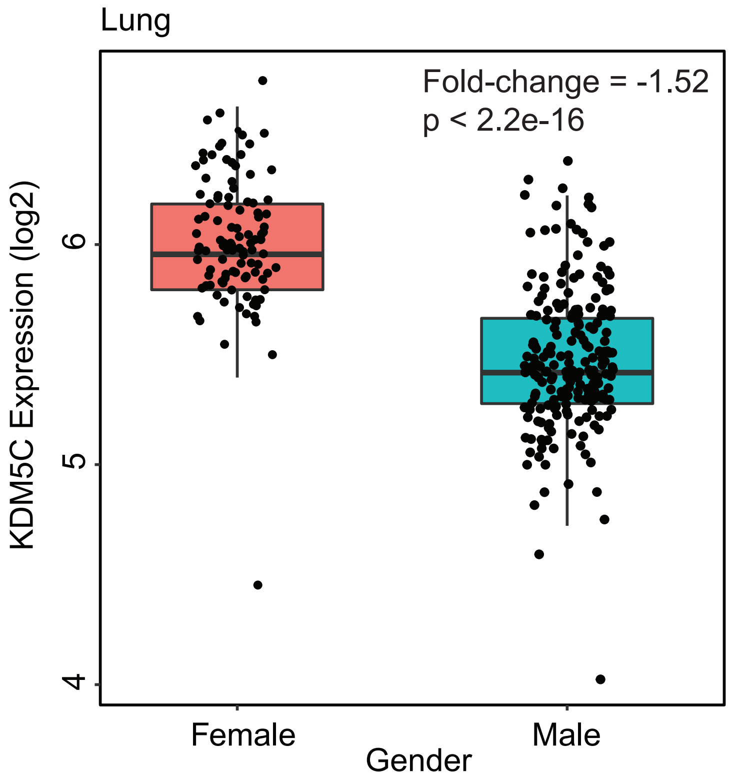

B

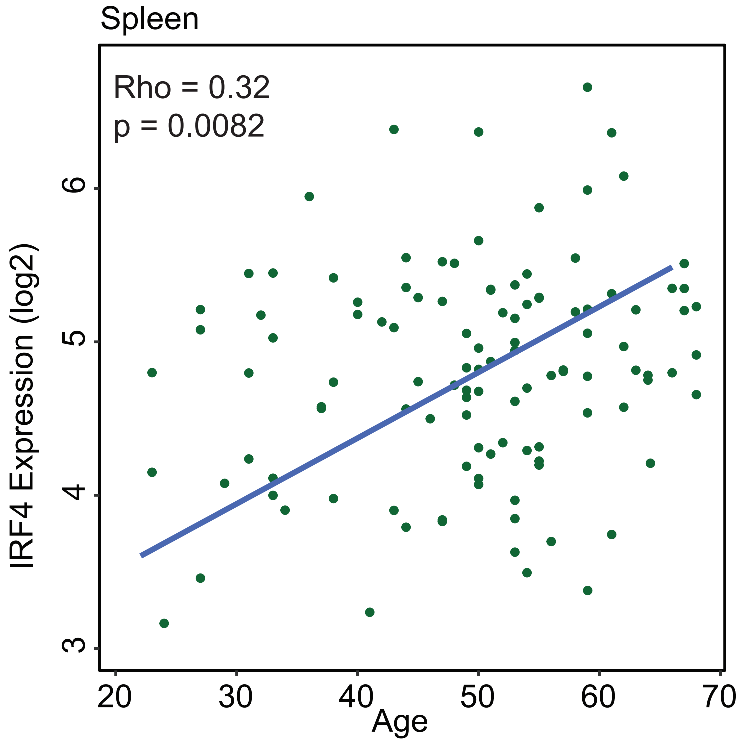

Figure S4. (A) Differential expression of KDM5C in lung tissue between females and males. (B) Significant correlation of IRF4 with age in splenic tissue.

Supplementary Table S1. Summary of sample size, detectable eRNAs, and trait-related eRNAs across tissues

Tissue	Sample Size	No. of eRNAs	Number of trait-related eRNAs
Adipose - Subcutaneous	385	5103	698
Adipose - Visceral (Omentum)	235	4836	380
Adrenal Gland	161	3877	44
Artery - Aorta	248	5540	272
Artery - Coronary	141	5301	96
Artery - Tibial	361	4519	725
Bladder	12	4881	0
Brain - Amygdala	83	2788	509
Brain - Anterior cingulate cortex (BA24)	100	3278	82
Brain - Caudate (basal ganglia)	135	3704	10
Brain - Cerebellar Hemisphere	120	8840	304
Brain - Cerebellum	147	12602	131
Brain - Cortex	133	4310	263
Brain - Frontal Cortex (BA9)	122	3643	131
Brain - Hippocampus	104	3116	212
Brain - Hypothalamus	104	3479	314
Brain - Nucleus accumbens (basal ganglia)	125	4111	3
Brain - Putamen (basal ganglia)	105	3383	1
Brain - Spinal cord (cervical c-1)	76	3365	1
Brain - Substantia nigra	72	2870	5
Breast - Mammary Tissue	221	6629	679
Cells - EBV-transformed lymphocytes	138	5992	82
Cells - Leukemia cell line (CML)	102	5931	0
Cells - Transformed fibroblasts	305	3996	91
Cervix - Ectocervix	6	2917	0
Cervix - Endocervix	5	3140	0
Colon - Sigmoid	175	5217	288
Colon - Transverse	207	5004	29
Esophagus - Gastroesophageal Junction	177	5233	497
Esophagus - Mucosa	338	4629	155
Esophagus - Muscularis	288	4578	57
Fallopian Tube	7	2348	0
Heart - Atrial Appendage	219	3877	53
Heart - Left Ventricle	274	2069	42
Kidney - Cortex	38	3567	0
Liver	141	3178	212
Lung	379	6496	120
Minor Salivary Gland	71	6704	31
Muscle - Skeletal	477	2819	210

Nerve - Tibial	328	8032	588
Ovary	112	7278	226
Pancreas	203	3379	25
Pituitary	126	6944	42
Prostate	120	7721	370
Skin - Not Sun Exposed	270	7464	1721
Skin - Sun Exposed	400	6485	288
Small Intestine - Terminal Ileum	106	6610	14
Spleen	121	6945	186
Stomach	209	3954	34
Testis	207	14232	84
Thyroid	365	7750	270
Uterus	93	7447	2257
Vagina	99	7680	582
Whole Blood	281	4185	377

Supplementary Table S2. Number of tissues and percentage of TFBS discovery rate for each TF.

TF	Number of Tissue in ENCODE	Percentage of co-expressed eRNA with TFBS
ADNP	1	97.15
AEBP2	1	97.78
AFF1	1	97.16
AHR	1	97.19
ARID2	1	96.38
ARNT	4	92.62
ATF1	2	91.55
ATF2	5	91.70
ATF3	6	93.51
ATF4	1	91.06
ATF7	4	91.51
BACH1	3	90.61
BATF	1	87.60
BCL11A	3	87.92
BCL11B	1	87.56
BCL6	1	88.90
BCL6B	1	90.51
BDP1	2	89.18
BHLHE40	6	89.70
CDC5L	1	83.45
CEBPB	7	83.37
CEBPG	3	81.26
CLOCK	1	76.33
CREB1	3	76.68
CREB3	1	74.46
CREB3L1	1	74.38
CREBBP	1	73.70
CREM	3	74.89
CSDE1	2	74.08
CTCF	142	99.46
CTCFL	1	69.56
CUX1	3	72.72
DEAF1	1	67.80
DPF2	3	65.60
E2F1	3	64.62
E2F4	5	62.83
E2F5	1	64.42
E2F6	2	63.40
E2F7	1	62.53
E2F8	3	60.18

E4F1	3	58.09
EGR1	4	58.57
EGR2	1	57.49
ELF1	5	57.77
ELF3	1	55.08
ELF4	2	55.58
ELK1	6	55.60
ELK4	2	57.32
EP300	20	99.31
ERF	1	55.23
ESR1	2	55.70
ESRRA	5	53.01
ETS1	5	55.06
ETS2	1	55.63
ETV1	1	56.06
ETV4	1	54.43
ETV5	1	54.75
ETV6	2	55.14
FEZF1	1	51.48
FOS	7	53.30
FOSL1	2	51.30
FOSL2	3	52.67
FOXA1	8	52.09
FOXA2	2	51.61
FOXA3	1	49.21
FOXJ2	1	49.61
FOXK2	5	48.79
FOXM1	3	42.18
FOXP1	1	44.17
FOXP2	2	41.92
GABPA	9	41.32
GATA1	2	38.81
GATA2	3	37.85
GATA3	3	38.88
GATA4	1	39.56
GATAD1	1	37.81
GATAD2A	2	37.69
GATAD2B	3	38.40
GFI1B	1	35.90
GLI2	1	37.48
GLI4	1	34.93
GLIS1	1	35.68
GLIS2	1	37.36
GMEB1	1	36.39

GMEB2	1	35.99
HES1	2	29.79
HHEX	1	29.10
HIC1	1	29.83
HINFP	1	27.08
HLF	1	27.83
HMBOX1	1	27.20
HNF1A	1	25.58
HNF4A	2	25.95
HNF4G	2	25.97
HOMEZ	1	24.84
ID3	1	24.64
IKZF1	3	24.73
IKZF2	1	25.18
IKZF3	1	24.64
IKZF5	1	24.84
INSM2	1	24.68
IRF1	1	24.79
IRF2	2	24.33
IRF3	4	23.71
IRF4	1	14.63
IRF5	1	14.42
IRF9	1	14.28
JUN	7	15.06
JUNB	2	14.84
JUND	10	14.55
KAT7	1	15.48
KDM5A	3	24.29
KDM5B	1	38.59
KLF1	2	14.00
KLF10	1	14.03
KLF11	1	13.93
KLF13	1	13.58
KLF16	3	13.46
KLF17	1	14.79
KLF4	1	14.84
KLF5	1	14.40
KLF7	1	14.33
KLF9	3	15.07
LCORL	1	14.96
MAFF	3	15.29
MAFG	1	14.86
MAFK	8	14.88
MAX	9	14.60

MAZ	8	14.01
MBD1	1	14.51
MBD2	2	15.08
MBD4	1	14.66
MEF2A	2	15.03
MEF2B	1	11.96
MEF2C	1	12.60
MEF2D	1	12.49
MEIS2	1	12.29
MGA	1	12.25
MITF	1	11.46
MIXL1	1	10.99
MLX	1	10.86
MNT	3	10.78
MTA1	2	9.30
MTA2	3	9.33
MXD4	1	8.44
MXI1	8	8.84
MYB	1	9.32
MYBL2	2	9.06
MYC	10	27.98
MYNN	2	8.09
MYRF	1	7.54
MZF1	1	7.37
NANOG	2	7.34
NCOA1	1	7.59
NCOA2	1	7.31
NCOA3	1	7.31
NCOR1	2	6.49
NEUROD1	2	6.02
NFATC1	1	6.43
NFATC3	2	5.87
NFE2	1	5.70
NFE2L1	1	5.83
NFE2L2	4	5.77
NFIL3	1	5.75
NFXL1	3	4.46
NFYA	3	4.22
NR2C1	2	4.17
NR2C2	4	3.70
NR2F1	3	4.09
NR2F2	2	4.12
NR2F6	2	3.88
NR3C1	4	3.88

NR4A1	1	4.20
NUFIP1	1	4.09
OSR2	1	3.90
OVOL3	1	3.25
PATZ1	1	3.88
PAX5	3	3.71
PAX8	2	3.54
PBX2	2	3.53
PBX3	1	3.43
PHF20	1	3.27
PKNOX1	4	3.06
POU2F2	2	2.28
POU5F1	3	2.26
PPARG	1	2.37
PRDM1	2	1.96
PRDM10	2	1.77
PRDM2	1	1.81
PRDM4	1	1.82
RARA	1	1.75
RBAK	1	1.41
RBPJ	1	1.10
RCOR1	8	1.03
RCOR2	1	1.21
RELA	5	1.17
RELB	1	1.19
REPIN1	1	0.39
RERE	1	0.32
REST	13	66.28
RFX1	3	0.30
RFX3	1	0.26
RFX5	9	0.26
RLF	1	0.03
RUNX1	1	0.02
RUNX3	1	99.30
RXRB	1	98.04
SALL2	1	94.29
SCRT1	1	91.83
SCRT2	1	91.44
SETDB1	2	93.17
SIX4	1	93.09
SIX5	4	89.43
SMAD1	2	90.44
SMAD2	1	91.08
SMAD4	1	90.70

SMARCC1	1	84.77
SMARCC2	3	83.22
SP1	8	82.42
SP2	4	79.76
SP3	1	81.49
SP4	1	79.28
SP5	1	78.07
SP7	1	78.72
SPI1	4	79.10
SREBF1	5	77.90
SREBF2	4	76.43
SRF	4	77.04
STAT1	3	76.78
STAT2	1	76.04
STAT3	3	76.96
STAT5A	2	75.31
TAL1	1	71.14
TBX21	1	65.15
TBX3	1	65.39
TCF12	5	65.78
TCF3	1	62.17
TEAD1	1	65.61
TEAD2	1	62.85
TEAD3	1	59.50
TEAD4	3	61.53
TFAP4	1	59.98
TFDP1	2	61.31
TFE3	1	59.69
TGIF2	1	60.45
THRA	1	59.09
THRB	1	55.84
TSHZ1	1	53.18
USF1	6	47.53
USF2	8	45.90
VEZF1	1	48.40
WT1	1	45.24
YBX1	4	44.92
YBX3	1	45.54
YY1	11	42.73
ZBED1	2	42.33
ZBTB1	2	42.55
ZBTB10	1	44.05
ZBTB11	3	43.07
ZBTB12	1	39.89

ZBTB17	1	38.73
ZBTB2	1	39.63
ZBTB20	1	39.91
ZBTB21	1	40.97
ZBTB25	1	38.95
ZBTB26	2	38.64
ZBTB33	7	39.29
ZBTB40	4	38.56
ZBTB44	1	39.18
ZBTB48	1	37.46
ZBTB49	1	38.12
ZBTB5	1	38.92
ZBTB6	1	38.82
ZBTB7A	3	37.35
ZBTB7B	1	38.35
ZBTB8A	2	38.89
ZEB1	3	39.87
ZEB2	2	38.88
ZFP3	1	36.39
ZFP37	1	37.06
ZFP41	1	35.59
ZFP64	1	36.40
ZFP69B	1	36.40
ZFP91	1	38.23
ZFX	6	36.57
ZHX1	2	37.21
ZHX2	2	35.89
ZIC2	1	34.66
ZKSCAN1	4	34.32
ZKSCAN8	2	34.53
ZMIZ1	1	31.88
ZNF10	1	32.05
ZNF112	1	31.99
ZNF114	1	32.12
ZNF121	1	31.80
ZNF132	1	31.17
ZNF133	1	30.22
ZNF138	1	31.44
ZNF140	1	30.80
ZNF143	4	32.12
ZNF148	2	31.49
ZNF155	1	31.54
ZNF157	1	29.96
ZNF16	1	30.23

ZNF169	1	30.03
ZNF174	1	27.25
ZNF175	2	26.72
ZNF18	1	26.55
ZNF184	2	27.00
ZNF189	1	27.38
ZNF19	1	26.69
ZNF197	1	26.72
ZNF2	1	26.15
ZNF202	1	25.62
ZNF205	1	25.12
ZNF207	3	26.35
ZNF211	1	25.04
ZNF214	1	25.69
ZNF217	2	25.70
ZNF221	1	26.18
ZNF23	1	25.95
ZNF239	1	24.78
ZNF24	5	25.19
ZNF248	1	24.64
ZNF26	1	25.53
ZNF263	2	23.70
ZNF266	1	23.56
ZNF274	7	23.49
ZNF280D	1	22.69
ZNF282	2	21.42
ZNF292	1	22.65
ZNF3	2	22.19
ZNF311	1	21.44
ZNF324	1	21.66
ZNF335	1	19.22
ZNF34	1	19.66
ZNF341	1	18.59
ZNF350	1	20.04
ZNF354B	1	18.50
ZNF354C	1	18.88
ZNF362	1	18.26
ZNF366	1	98.69
ZNF37A	1	98.52
ZNF384	4	98.44
ZNF391	1	97.51
ZNF394	1	97.28
ZNF398	1	96.62
ZNF404	1	95.92

ZNF407	1	92.63
ZNF416	1	90.22
ZNF423	1	91.29
ZNF426	1	91.09
ZNF433	1	91.34
ZNF444	1	89.03
ZNF449	1	90.69
ZNF473	1	89.26
ZNF48	2	88.21
ZNF488	1	88.89
ZNF501	1	90.32
ZNF507	2	89.64
ZNF510	1	87.76
ZNF512	1	87.73
ZNF512B	1	86.62
ZNF513	1	85.45
ZNF514	1	85.51
ZNF521	1	83.64
ZNF529	1	81.46
ZNF530	1	80.25
ZNF544	1	74.27
ZNF547	1	74.37
ZNF548	1	75.57
ZNF549	1	74.60
ZNF555	1	74.93
ZNF558	1	75.24
ZNF560	1	77.58
ZNF561	1	75.21
ZNF571	1	74.15
ZNF574	1	72.63
ZNF579	1	71.78
ZNF580	2	71.90
ZNF584	1	72.52
ZNF589	1	65.15
ZNF592	3	64.83
ZNF596	1	65.92
ZNF600	1	65.80
ZNF610	1	63.98
ZNF614	1	62.67
ZNF621	1	62.33
ZNF624	1	62.18
ZNF629	1	55.98
ZNF639	2	57.27
ZNF644	2	57.02

ZNF645	1	52.78
ZNF652	1	55.30
ZNF658	1	54.76
ZNF660	1	54.68
ZNF664	1	55.22
ZNF670	1	53.86
ZNF677	1	52.43
ZNF680	1	53.26
ZNF687	2	50.79
ZNF692	1	49.84
ZNF7	1	50.18
ZNF701	1	47.77
ZNF704	1	48.19
ZNF707	1	45.36
ZNF76	1	44.36
ZNF766	1	45.33
ZNF768	1	43.43
ZNF770	1	46.77
ZNF776	1	45.17
ZNF777	1	44.66
ZNF785	1	44.76
ZNF791	1	42.31
ZNF83	1	41.59
ZNF843	1	41.45
ZSCAN16	1	40.68
ZSCAN18	1	41.28
ZSCAN21	1	41.27
ZSCAN23	1	41.90
ZSCAN29	2	40.47
ZSCAN30	1	40.44
ZSCAN4	1	39.54
ZSCAN5A	1	39.49
ZSCAN5C	1	37.27
ZSCAN9	1	39.96
ZXDB	1	41.24
